# Modulation of Hemostatic and Inflammatory Responses by *Leptospira* Spp.

**DOI:** 10.1371/journal.pntd.0004713

**Published:** 2016-05-11

**Authors:** Mônica L. Vieira, Clément Naudin, Matthias Mörgelin, Eliete C. Romero, Ana Lucia T. O. Nascimento, Heiko Herwald

**Affiliations:** 1 Department of Clinical Sciences, Lund, Division of Infection Medicine, Lund University, Lund, Sweden; 2 Centro de Biotecnologia, Instituto Butantan, Sao Paulo, Sao Paulo, Brazil; 3 Centro de Bacteriologia, Instituto Adolfo Lutz, Sao Paulo, Sao Paulo, Brazil; Universidad Peruana Cayetano Heredia, PERU

## Abstract

Leptospirosis is a worldwide spread zoonotic and neglected infectious disease of human and veterinary concern that is caused by pathogenic *Leptospira* species. In severe infections, hemostatic impairments such as coagulation/fibrinolysis dysfunction are frequently observed. These complications often occur when the host response is controlled and/or modulated by the bacterial pathogen. In the present investigation, we aimed to analyze the modulation of the hemostatic and inflammatory host responses by the bacterial pathogen *Leptospira*. The effects of leptospires and their secreted products on stimulation of human intrinsic and extrinsic pathways of coagulation were investigated by means of altered clotting times, assembly and activation of contact system and induction of tissue factor. We show that both extrinsic and intrinsic coagulation cascades are modulated in response to *Leptospira* or leptospiral secreted proteins. We further find that the pro-inflammatory mediator bradykinin is released following contact activation at the bacterial surface and that pro-coagulant microvesicles are shed from monocytes in response to infection. Also, we show that human leptospirosis patients present higher levels of circulating pro-coagulant microvesicles than healthy individuals. Here we show that both pathways of the coagulation system are modulated by leptospires, possibly leading to altered hemostatic and inflammatory responses during the disease. Our results contribute to the understanding of the leptospirosis pathophysiological mechanisms and may open new routes for the discovery of novel treatments for the severe manifestations of the disease.

## Author Summary

Leptospirosis is one of the most relevant and spread zoonotic and neglected infectious diseases affecting humans and other mammals, and is caused by pathogenic bacteria of the genus *Leptospira*. During infectious diseases, when bacterial pathogens control and/or modulate the host response, impaired hemostasis and inflammation are frequently observed. Here we studied the effects of leptospires and their secreted products on stimulation of human intrinsic and extrinsic pathways of coagulation, showing that both coagulation cascades are modulated in response to *Leptospira* or leptospiral secreted proteins. We further find that activation of the coagulation cascades culminates in the release of the pro-inflammatory mediator bradykinin and noted an induction of pro-coagulant microvesicles. These findings contribute to a better understanding of the local and systemic hemostastic complications during leptospirosis. Collectively, our results show how leptospires can affect host responses, possibly leading to altered host responses during the disease and giving rise to the leptospirosis symptomatology.

## Introduction

Leptospirosis is an infectious disease caused by pathogenic bacteria of the genus *Leptospira* [1, 2]. In humans, infections are mainly acquired through contact with wild or domestic infected animals or exposure to contaminated soil or water [3, 4]. It is estimated that more than 500,000 cases of leptospirosis occur annually world-wide [5].

Leptospires enter the host mainly via sodden or damaged skin or mucosa, followed by rapid dissemination through the blood stream. In the acute phase, or leptospiremia, bacteria may multiply in the circulation and spread into the surrounding tissue, being the kidneys and liver the preferential colonization sites. Soon after the host has mounted a specific immunological response, bacteria are cleared from blood, characterizing the immune or convalescent phase [2]. Infections can trigger a wide spectrum of clinical symptoms, varying from subclinical to severe manifestations. The most severe conditions known as Leptospirosis Pulmonary Hemorrhagic Syndrome and Weil’s disease, the last characterized by jaundice, hypotension, meningitis, kidney and multiple organ failure and hemorrhages, result in mortality rates up to 70% and 15% respectively [2, 6]. The mechanisms of pathogenicity and virulence of the leptospires are still to be elucidated and the origin of pathophysiological leptospirosis symptoms and severity of disease remain virtually unknown [7–9].

During infection, inflammatory mediators from the microbe and/or host can induce complications by modulating the hemostatic equilibrium between the pro-coagulant and anticoagulant status of the host [10]. The coagulation cascade can be divided into two pathways, of which the extrinsic pathway is induced by tissue factor (TF) exposure and/or release and considered as the primary pathway coagulation [11]. The intrinsic pathway of coagulation, also referred to as the contact system, seems to play a secondary role in the processes. However, its activation can lead to a pro-inflammatory state via the release of bradykinin (BK) [12–14]. In severe bacterial infections, dysregulation of the host innate immune system and hemostasis can contribute to a fatal outcome. Notably, these complications often involve both pathways of the coagulation system [15].

We have previously reported that *Leptospira* are able to modulate the human fibrinolytic system. This interaction involves the capturing of human plasminogen on the surface of the microorganism, leading to increased pathogen-associated plasmin activity [16]. Leptospires also promote an imbalance of the normal fibrinolysis by enhancing the availability of plasminogen activators [17]. Bacterial membrane-associated plasmin can favor bacterial penetration, and also enhance immune evasion activity, a critical condition during hematogenous dissemination [17–19].

Some studies report ongoing fibrinolysis, activation of coagulation, impaired anticoagulation and thrombocytopenia during leptospirosis, while the involvement of disseminated intravascular coagulation (DIC) is controversial [20–24]. As leptospirosis can lead to serious hemorrhagic syndromes and to date no mechanism responsible for this is known, it is generally believed that impaired hemostasis through coagulation/fibrinolysis dysfunction might be involved. Thus, the present investigation aims to further decipher the role of these systems in leptospirosis.

## Methods

### Patient serum samples

Confirmed leptospirosis human serum samples were obtained from Instituto Adolfo Lutz sera collection, Sao Paulo, Brazil, and were donated for research purposes. We had no access to any patient data. The use of serum samples from Adolfo Lutz sera collection was waived from official ethics approval by the Ethical Committee for Human Research of Universidade de Sao Paulo, which rules that this work does not involve procedures regulated by CONEP/Brazil n° 466/2012. We used paired samples from the same patients at the early phase (MAT negative, negative microscopic agglutination test) and convalescent phase of leptospirosis (MAT positive). The MAT was performed according to Faine et al. [3]. In brief, an array of serovars of *Leptospira* spp. as antigens were employed. A laboratory-confirmed case of leptospirosis was defined by a four-fold microagglutination titer rise between paired serum samples. MAT was considered negative when the titer was below 100.

### Bacteria strains and culture conditions

Virulent low-passage *L*. *interrogans* serovar Copenhageni strain L1-130, non-virulent culture-attenuated *L*. *interrogans* serovar Copenhageni, and saprophytic *L*. *biflexa* serovar Patoc were kindly provided by Dr. Mathieu Picardeau (Institute Pasteur, France). Leptospires were cultured at 28°C in Elinghausen-McCullough-Johnson-Harris (EMJH) medium (BD, Difco) supplemented with 10% *Leptospira* enrichment EMJH (BD, Difco), 0.3 g/L peptone (BD, Difco) and 0.2 g/L meat extract (Sigma-Aldrich).

### Blood and plasma

Blood samples were drawn from healthy volunteers in Vacutainer tubes (Becton Dickinson) containing 1/9 volume 109 mM sodium citrate, pH 7.4. Blood was used immediately or centrifuged for plasma separation. Informed consent was obtained from all healthy blood donors.

### Clotting assays

Mid-log phase culture Leptospires were enumerated, washed twice with HEPES buffer (115 mM NaCl, 1.2 mM CaCl_2_, 1.2 mM MgCl_2_, 2.4 mM K_2_HPO_4_, 20 mM HEPES) and resuspended in HEPES buffer containing 50 μM ZnCl_2_ (HEPES-ZnCl_2_). 300 μL of the cells suspensions (5x10^9^, 1x10^9^, 1x10^8^ or 1x10^7^ cells/mL) or mid-log phase culture supernatants were added to the same volume of blood or plasma. Fresh culture medium or buffer alone were employed as controls. After incubation for 0.5, 1, 2 or 4 h at 37°C, 50 μL 30 mM CaCl_2_ were added to 100 μL of the samples, and the recalcification clotting times (i.e. after calcium addition) were measured in a semi-automatic ball coagulometer (MC10plus, Amelung).

Alternatively, as a strategy to analyze the plasma after bacterial interaction with coagulation factors, bacteria were removed by centrifugation (4000 x *g*, 15 min) after the incubation in plasma and the recalcification clotting times of the supernatants were measured. The remaining bacterial pellets were resuspended in 300 μL HEPES-ZnCl_2_, mixed with fresh plasma, and the recalcification clotting times immediately measured.

To measure aPTT (activated partial thrombin time), PT (prothrombin time), and TCT (thrombin clotting time), 1x10^9^ bacteria/mL were washed and resuspended in buffer. The samples were mixed with the same volume of human plasma, and after 30 min incubation at 37°C, bacteria were removed by centrifugation. To trigger the clotting reactions, 100 μL of the resulting supernatants were mixed with 20 μL PT (TriniCLOT PT, Trinity Biotech) or 50 μL TCT reagents (3.4 IU/mL thrombin), and the times for plasma to clot were recorded. Alternatively, 100 μL of the samples were mixed with 50 μL aPTT reagent (DAPTTIN TC—Technoclone) and after 200 s incubation, 50 μL 30 mM CaCl_2_ were added to initiate the clotting reaction.

### Peripheral blood mononuclear cells (PBMCs) stimulation and pro-coagulant activity

Human PBMCs were purified from citrated blood using Lymphoprep gradient (AXIS-SHIELD), according to the manufacturer instructions. Equal volumes of PBMCs suspensions (5x10^6^ cells/mL) and leptospires (2x10^7^/mL) or bacteria culture supernatants were mixed and incubated at 37°C for 2 h. Buffer alone or EMJH were used as controls. The PBMCs were pelleted by centrifugation and resuspended in 600 μL normal plasma or FVII-deficient plasma diluted 50% in HEPES-ZnCl_2_. Normal plasma was used to verify the general status of the coagulation, while FVII-deficient plasma as a control to exclude the activity of the TF/extrinsic pathway of coagulation. The recalcification clotting times were measured by addition of 50 μL 30 mM CaCl_2_ to 100 μL of sample. Alternatively, the aPTTs were measured in order to verify the status of the intrinsic (contact system) and common pathways of coagulation. aPTTs were obtained by addition of 50 μL aPTT reagent to 50 μL of sample followed by 200 s incubation and addition of 50 μL 30 mM CaCl_2_.

### Coagulation factors activity assays

To evaluate the assembly and activation of the contact system at the bacterial surface, we used the chromogenic substrate S-2302, specific for plasma kallikrein (PK). To further assess the activity of downstream activation of the final common coagulation pathway, we used the chromogenic substrates specific for factor Xa (FXa) and thrombin (S-2765 and S-2238, respectively). All substrates were purchased from Chromogenix. 3x10^9^ bacteria were washed and resuspended in 300 μL HEPES-ZnCl_2_. Bacterial suspensions were added to equal volumes of plasma with or without 100 μg/mL of the peptide H-D-Pro-Phe-Arg-chloromethylketone, a specific inhibitor of the contact system by interfering with factor XII (FXII) and PK activities [25], (N-1210, Bachem) (or buffer, as control). After incubation at 37°C for 30 min, bacteria were washed three times and resuspended in 450 μL buffer. Chromogenic substrates (50 μL of 4 mM solution) were added to 150 μL aliquots of bacterial suspensions. After 30 min incubation at 37°C, cells were removed and the optical densities (405 nm) of the supernatants were measured.

### Elution and detection of coagulation factors bound to the bacterial surface

*Leptospira* suspensions (3x10^8^ in 300 μL HEPES-ZnCl_2_) were mixed with equal volumes of human plasma with or without 100 μg/mL N-1210. After 30 min incubation at 37°C, bacteria were pelleted and extensively washed. To elute the bacteria-bound proteins, 100 μL 0.1 M glycine (pH 2.0) were added to the bacterial pellets. Bacteria were removed by centrifugation and after addition of 10 μL 1 M Tris pH 7.4, the supernatants were subjected to 10% SDS-PAGE. The gels were transferred to membranes and probed with antibodies against (FXII) or high molecular weight kininogen (HK). After incubation with HRP-conjugated antibodies, reactivity was detected by chemiluminescence.

### Bradykinin (BK) assay

Bacterial suspensions (1x10^9^ cells/mL in HEPES-ZnCl_2_) were incubated with equal volume of normal or kallikrein-deficient plasma for 30 min at 37°C. Some samples received the addition of N-1210 (100 or 25 μg/mL) or the cysteine proteinase inhibitor E-64 (100 μg/mL). Bacteria were washed, resuspended in buffer, incubated for 30 min at 37°C and centrifuged. The supernatants were collected for BK release determination by an ELISA kit (ab136936, Abcam).

### TF and TFPI assays

Quantification of TF and TFPI from human sera samples were performed using commercial ELISA kits (ab108903 and ab108904, Abcam), according to the manufacturer instructions. For TF and TFPI determinations, we used paired serum samples from 10 and 12 patients, respectively.

### Microvesicles (MVs) isolation

Pooled leptospirosis patients sera or normal human sera (200 μL) were centrifuged (20,800 x *g*, 30 min). Supernatants were disposed and the MVs were washed two times in 900 μL PBS. Following centrifugation (20,800 x *g*, 30 min), 800 μL of supernatants were removed, and the MVs were resuspended in the remaining buffer. For MVs isolation, we used paired sera from 10 patients, pooling 5 MAT negative and 5 MAT positive for two independent isolations.

For the isolation of PBMCs-derived MVs, the cells were stimulated with leptospires or culture supernatants for 24 h at 37°C. After centrifugation to remove the cells and bacteria, the supernatants were subjected to MVs isolation as described above.

### Immunoelectron microscopy

TF was labeled with colloidal gold as described earlier [26]. MVs preparations were mixed with gold-labeled TF and processed for negative staining [27].

## Results

### *Leptospira* bacteria and culture supernatants induce pro-coagulant activity in human blood and plasma

**Fig 1:**
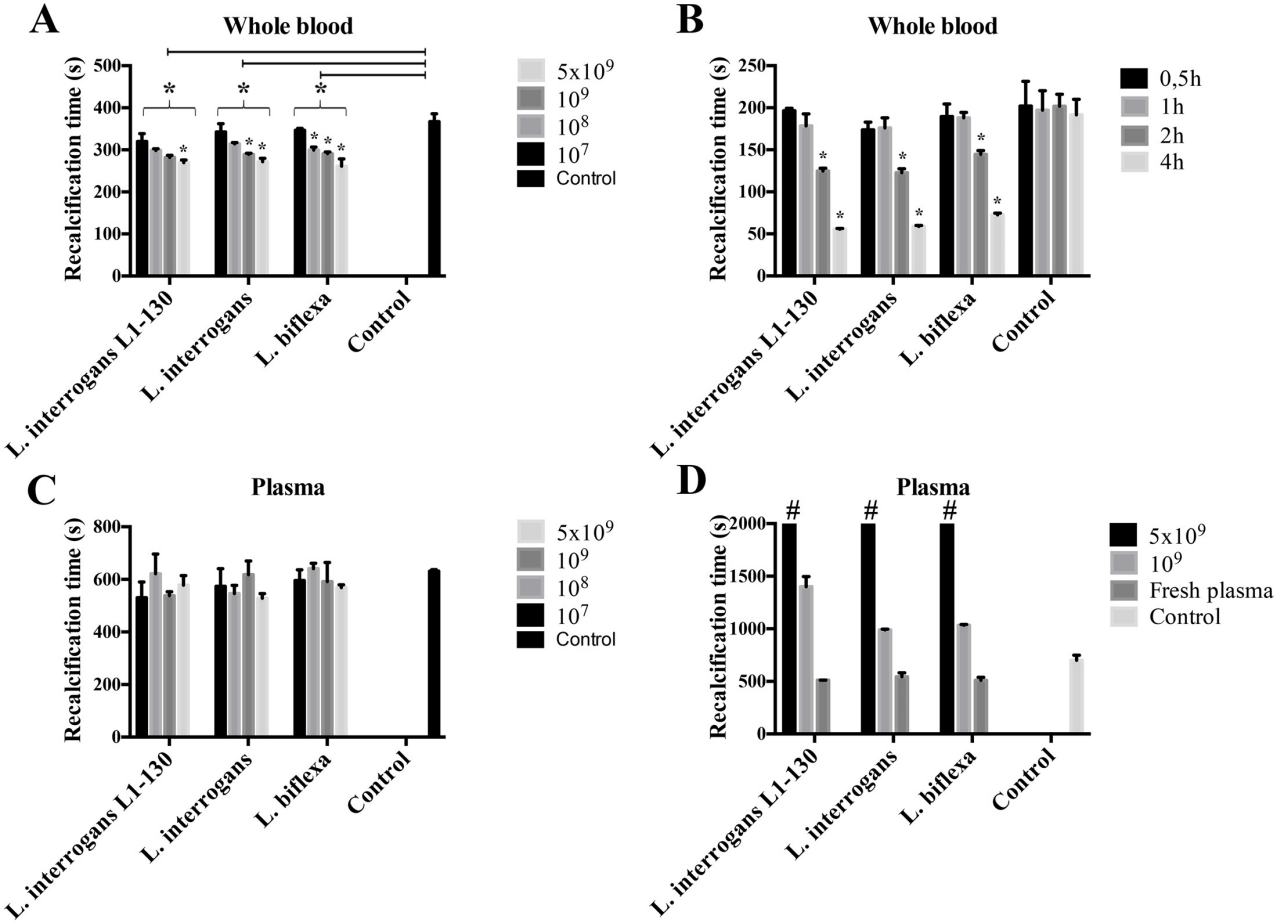
*Leptospira* bacteria trigger pro-coagulant activity in human blood. Virulent *L*. *interrogans* serovar Copenhageni L1-130, culture-attenuated *L*. *interrogans* serovar Copenhageni, and saprophytic *L*. *biflexa* were incubated with citrated human blood at different bacterial concentrations (*panel A*) or increasing incubation intervals (*panel B*) and the clotting times after calcium addition were determined in a coagulometer. *Panel C*, the recalcification times of human plasma incubated with different bacterial concentrations in human plasma was determined. *Panel D*, bacteria were incubated with human plasma for 30 min and removed by centrifugation. After the addition of calcium, the coagulative state of the remaining plasma samples was measured. Plasma incubated in the absence of bacteria and buffer alone were used as control in all experiments. The bars represent the means ± standard deviation of four replicates and are representative of three independent experiments. *P > 0.05: in *panel A*, all the bacterial samples were statistically significant compared to the control. The different strains employed at different concentrations also had the significance tested in comparison to the highest concentration (5x10^9^). In *panel B*, is shown the significance between the different incubation times of each strain. # no clotting after 2000 s.

To analyze whether leptospires can influence the human coagulation cascade, bacteria were incubated with citrated human blood and the recalcification clotting times were determined. The two pathogenic strains (virulent and culture attenuated *L*. *interrogans*) and the non-pathogenic strain (*L*. *biflexa*) reduced to the same extent the clotting times in a dose and time-dependent manner (Fig 1A and 1B). Notably, incubation of bacteria with human plasma instead of human blood did not alter the recalcification clotting times (Fig 1C). Together these data suggest that clotting is mainly triggered by activation of the extrinsic pathway of coagulation, as it is dependent on the cellular components of human blood.

In the next experiments bacteria were incubated with human plasma for 30 min and then removed by centrifugation. After calcium addition to the remaining plasma a dramatic and strain-independent increase of the recalcification clotting times was noted at a *Leptospira* load of 1x10^9^ bacteria/mL and when concentrations were 5x10^9^ bacteria/mL clot formation was completely prevented (Fig 1D). These findings suggest that a depletion of coagulation factors possibly via binding to the bacteria surface or degradation/consumption is responsible for the impairment of normal clotting.

**Fig 2:**
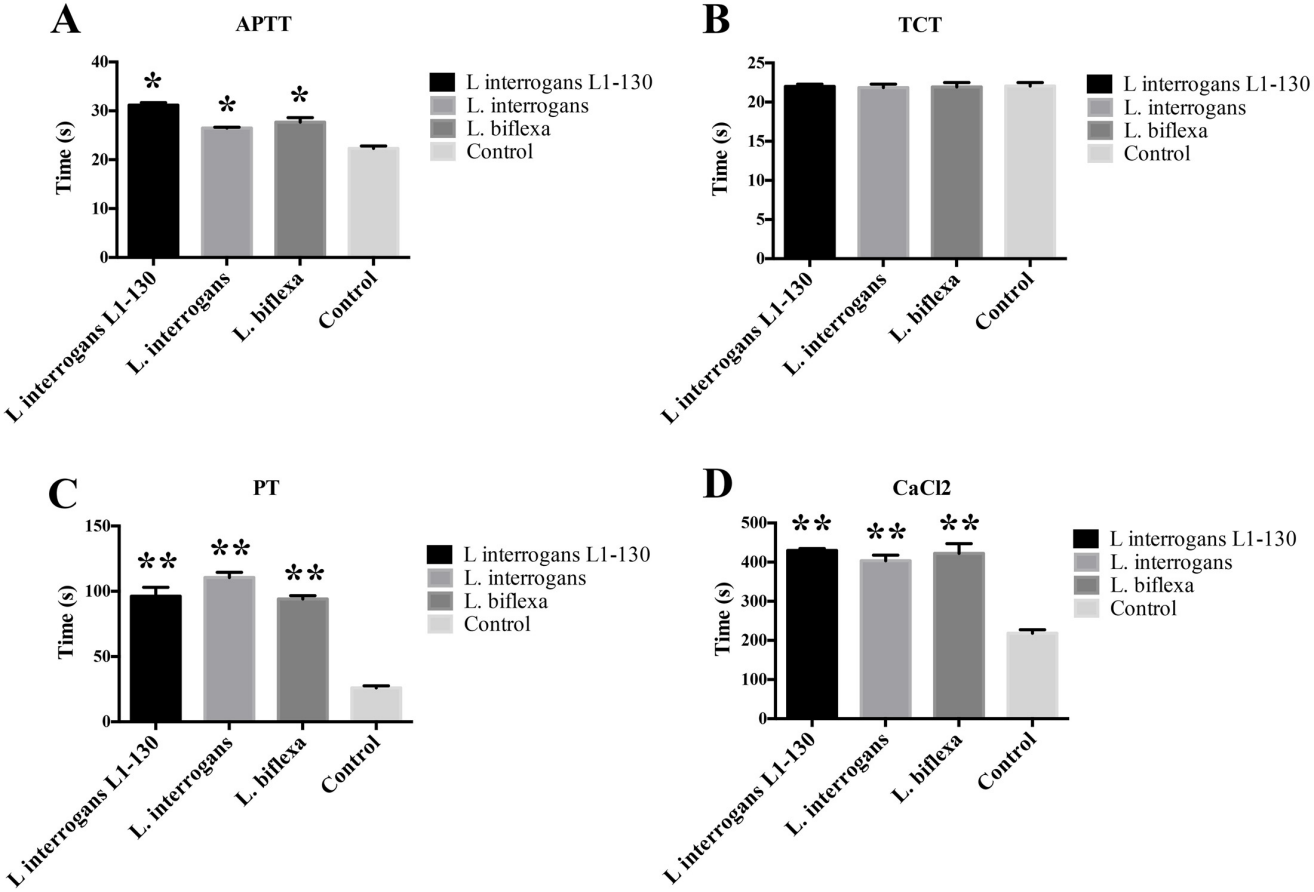
*Leptospira* interferes with aPTT and PT of normal human plasma. Virulent *L*. *interrogans* serovar Copenhageni L1-130, culture-attenuated *L*. *interrogans* serovar Copenhageni, and saprophytic *L*. *biflexa* (100μL of 1x10^9^ bacteria/mL) were mixed with the same volume of human plasma and the aPTT (A), TCT (B), and PT (C) were determined in a coagulometer as detailed in M&M section. The recalcification clotting times were performed for comparative purpose (D). Buffer was used as control in all experiments. The bars represent the means ± standard deviation of four replicates and are representative of three independent experiments. *P > 0.05 and **P > 0.005.

To better understand the coagulation disturbances caused by *Leptospira*, bacteria (1x10^9^/mL) were mixed with human plasma, removed by centrifugation and clotting times measuring activation of the extrinsic, intrinsic, and common pathway of coagulation were determined in the remaining supernatants. Prolonged aPTT and PT, but not TCT, values were observed for all three strains as compared to plasma samples incubated with buffer alone (Fig 2A–2C). We additionally tested whether *Leptospira* strains influence the aPTTs, PTs, or TCTs, showing that also under these conditions normal clotting was impaired (Fig 2D). Together our data suggest that coagulation is induced by an up-regulation of TF at cellular level, while the interaction of the bacteria with coagulation factors decelerates the time to clot formation.

When bacterial culture supernatants were used to modulate clotting in human blood and plasma, results similar to the ones obtained with the bacteria were observed. Supernatants from the three strains reduced the recalcification clotting times of blood and plasma (S1 Fig). Thus, leptospires have both secreted and surface-associated products which are able to modulate the coagulant state in human blood and plasma.

### Human PBMCs upregulate TF in response to *Leptospira* and its secreted products

**Fig 3:**
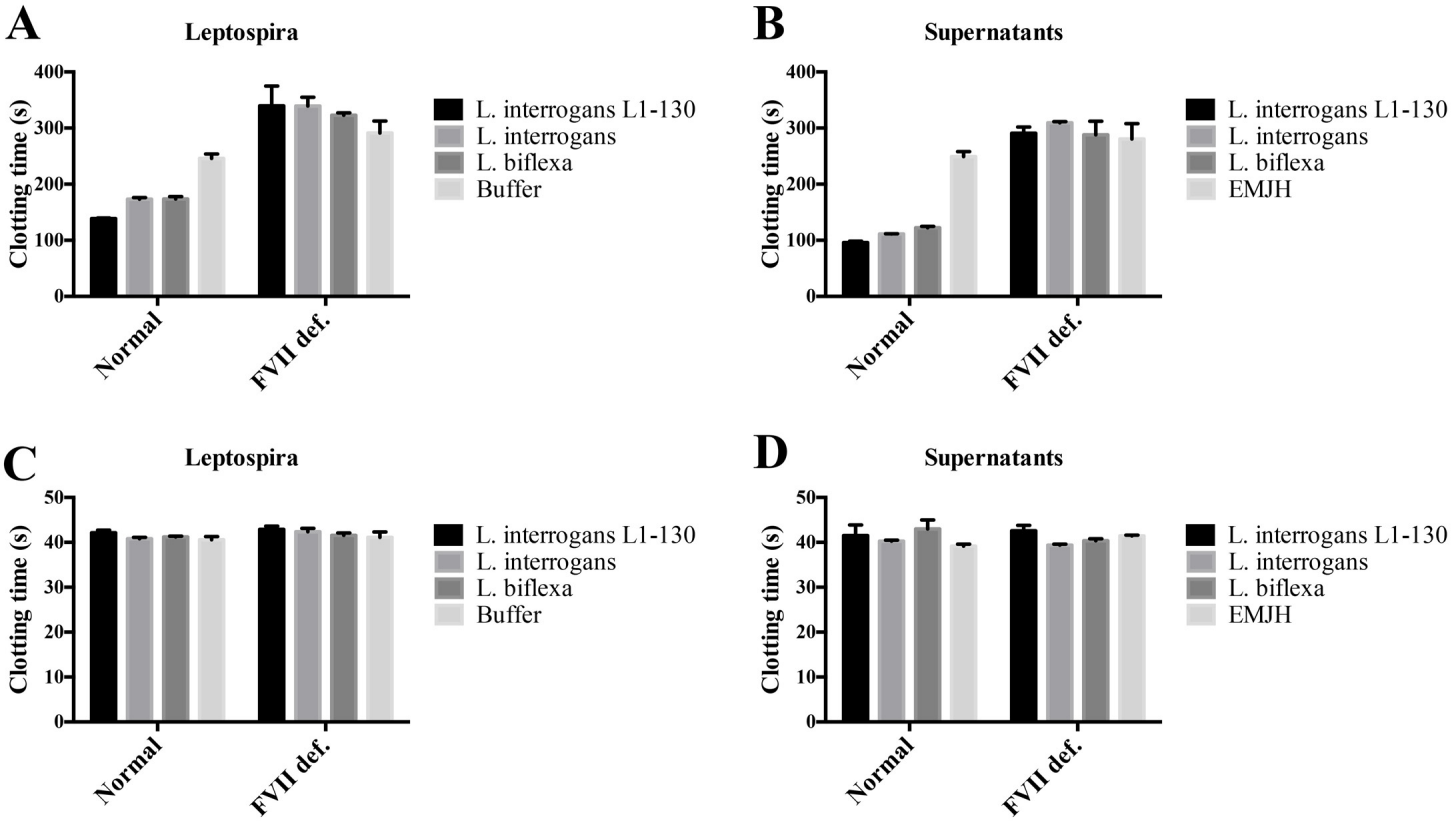
Leptospires and their secreted products induce pro-coagulant activity in human PBMCs. Human PBMCs were isolated and incubated with virulent *L*. *interrogans* serovar Copenhageni L1-130, culture-attenuated *L*. *interrogans* serovar Copenhageni, and saprophytic *L*. *biflexa* (*panel A* and *C*) or their respective culture supernatants (*panel B* and *D*). After incubation, PBMCs were washed and the ability of the cells suspensions to induce clot formation on fresh human plasma was determined by measuring recalcification clotting times in the absence (*panel A* and *B*) or presence of the aPTT reagent (*panel C* and *D*). Cells incubated in buffer alone or fresh culture medium were used as controls. The bars represent the means ± standard deviation of three replicates and are representative of two independent experiments.

Having shown that *Leptospira* and culture supernatants can trigger pro-coagulant activities in human blood, we next investigated whether this effect is caused by an up-regulation of TF. Bacteria and culture supernatants were incubated with isolated human PBMCs. Treated and untreated cells were added to fresh human plasma and the recalcification clotting times were determined. All three strains and their respective supernatants rendered PBMCs into a pro-coagulant state. The data further reveal that clotting is controlled by the extrinsic pathway of coagulation, because only decreased times were measured when normal but not FVII-deficient plasma was used (Fig 3A and 3B). When stimulated or non-stimulated cells were added to normal or FVII-deficient plasma and aPTTs measured to verify the status of the intrinsic pathway of coagulation, no differences were noted (Fig 3C and 3D), confirming that the pro-coagulant activity is mainly caused by the up-regulation of TF on PBMCs.

### Contact system recruitment, HK processing, and BK release at the bacterial surface

**Fig 4:**
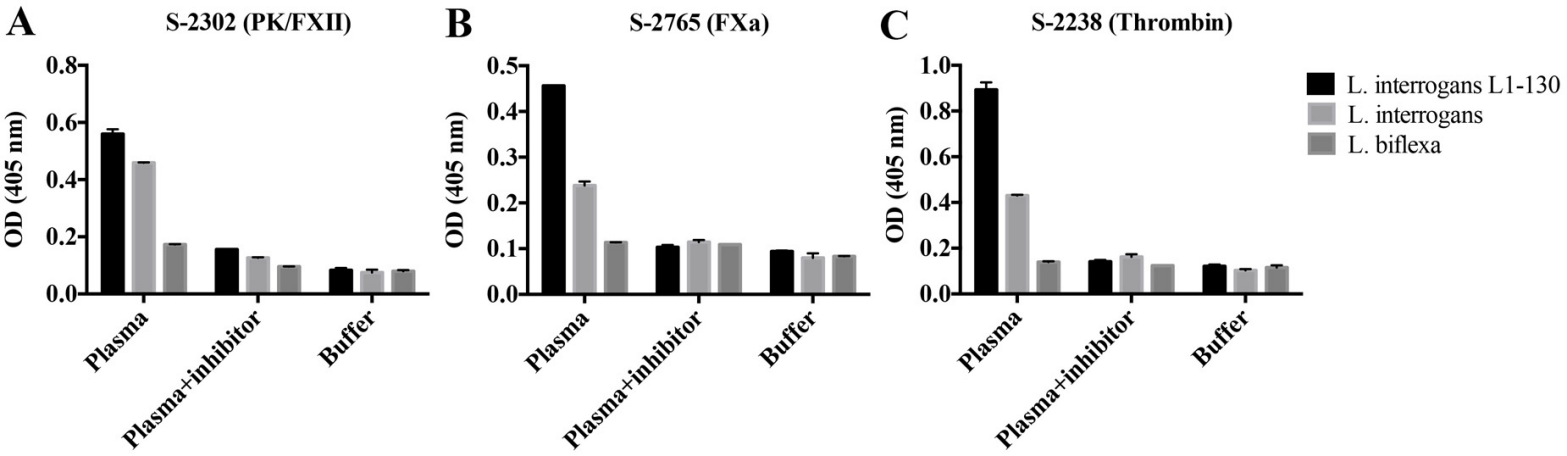
Assembly and activation of coagulation factors at the surface of *Leptospira*. Virulent *L*. *interrogans* serovar Copenhageni L1-130, culture-attenuated *L*. *interrogans* serovar Copenhageni, and saprophytic *L*. *biflexa* were incubated with human plasma, washed, and resuspended in buffer. The binding and activity of coagulation factors associated to the bacterial membrane were assessed using specific chromogenic substrates: S-2302 (PK and FXII) (A), S-2765 (FXa) (B) and S-2238 (thrombin) (C). As control bacteria and plasma were incubated in the presence of the contact system inhibitor N-1210. Bacteria incubated in buffer alone were used as negative controls. The bars represent the means ± standard deviation of three replicates and are representative of three independent experiments.

Our results showing that addition of *Leptospira* to human plasma impairs the intrinsic pathway of coagulation, implies an interaction of the bacteria with factors of the contact system. To address this issue, bacteria were incubated with human plasma, washed, and the binding and activation of contact factors at the bacterial surface was assessed by specific chromogenic substrates. The assembly and activation of the contact system occurs at the surface of the three strains tested and resulted in not only a hydrolysis of the substrates specific for plasma kallikrein/FXII (Fig 4A), assessing the status of the intrinsic pathway of coagulation, but also of substrates specific for FXa (Fig 4B) and thrombin (common pathway) (Fig 4C). Interestingly, activation seems to correlate with the virulence of the strains tested, as *L*. *interrogans* L1-130 was the most potent followed by the non-virulent culture-attenuated *L*. *interrogans* and the non-pathogenic *L*. *biflexa* strains. When the experiments were conducted in the presence of a specific inhibitor of FXII and PK, peptide N-1210 [25], a strong decrease of the PKa activity was measured and also the activity of FXa and thrombin was significantly reduced. Together the results show that leptospires are able to assemble and activate the contact system at their surface, leading to an activation of the up-stream coagulation factors i.e. FXa and thrombin. The data further suggest that the extent of activation may correlate with the virulence of these strains.

**Fig 5:**
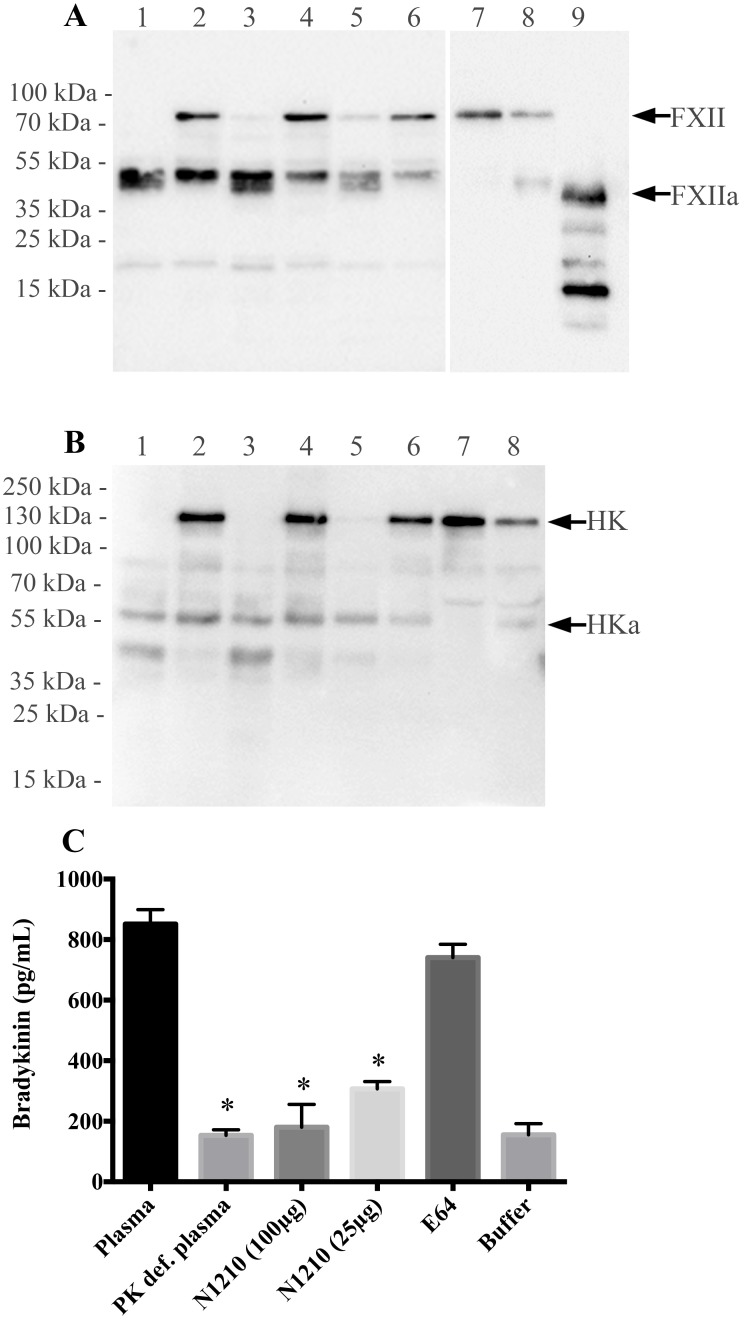
Binding and activation of FXII and HK processing at the surface of *Leptospira*. Bacteria were incubated with human plasma in the presence or absence of the contact system inhibitor N-1210 and washed. Bound proteins were eluted from the bacteria and subjected to Western blot analysis using antibodies against FXII (A) and HK (B). Human plasma (lane 7) and human plasma activated with kaolin (lane 8) served as controls. Lanes 1 and 2: virulent *L*. *interrogans* serovar Copenhageni. Lanes 3 and 4: culture-attenuated *L*. *interrogans* serovar Copenhageni. Lanes 5 and 6: *L*. *biflexa*. Experiments were performed in the absence (lanes 1, 3, and 5) or presence (lanes 2, 4 and 6) of N-1210. Lane 9: purified FXIIa. The arrows indicate full length FXII and HK and their respective cleavage products (FXIIa and HKa). The binding experiments were performed twice with similar results. (C) Virulent *L*. *interrogans* serovar Copenhageni were incubated with human plasma, washed, and resuspended in buffer. After incubation, bradykinin contents in the supernatants were measured by ELISA. Peptide N-1210 was added to the reaction mixture at 25 μg/mL and 100 μg/mL and the cysteine proteinase inhibitor, E64 (100 μg/mL), was used as control. To determine background levels, bacteria were incubated with plasma kallikrein (PK) deficient plasma or buffer alone. The bars represent the media ± standard deviation of three replicates and are representative of three independent experiments. *P > 0.005 in comparison to Plasma samples.

To further analyze the binding and processing of contact proteins at the leptospiral surface, bacteria were incubated in human plasma in the absence or presence of N-1210. Bound proteins were eluted and subjected to Western blot analysis using antibodies against FXII and HK. Fig 5A depicts FXII is recovered in its active form from the bacterial surface of all strains when plasma was incubated in the absence of peptide N-1210, while in the presence of the inhibitor, activation of FXII was notably prevented. When analyzing the degradation pattern of HK under the same experimental conditions, similar findings were obtained, implying that also HK is processed at the bacterial surface of the three strains (Fig 5B).

To test whether HK cleavage is followed by the release of BK, the virulent *L*. *interrogans* strain and human plasma were incubated for 30 min and the release of BK from the bacterial surface after was measured after a washing step. Incubation of bacteria with normal plasma resulted in a massive BK release, which was not seen when PK-deficient plasma was used or plasma was replaced by buffer (Fig 5C). Addition of N-1210 during the plasma incubation step resulted in a dose-dependent inhibition of BK release, while co-incubation with a cysteine proteinase inhibitor (E64) had no significant effect. Taken together, these results indicate that the contact system is activated at the bacterial surface leading to a subsequent activation of further coagulation factors and the generation of BK.

### Increased TF levels in leptospirosis patients’ samples

**Fig 6:**
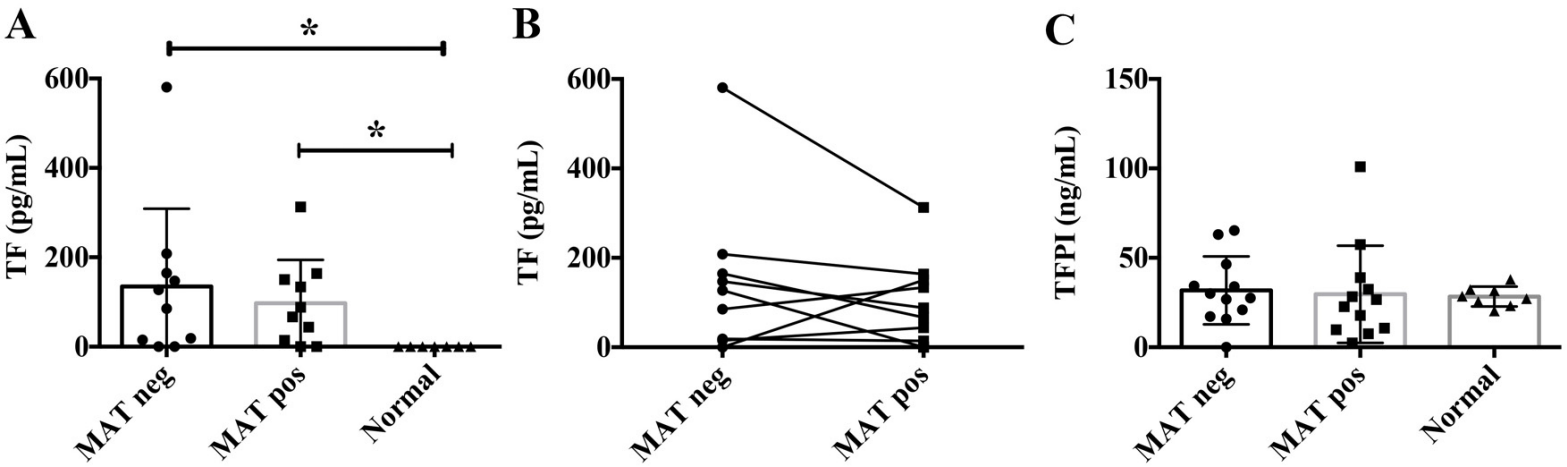
Measurements of TF and TFPI levels in sera from patients diagnosed with leptospirosis. TF (A and B) and TFPI (C) were quantified by commercial capture ELISA kits in the sera of leptospirosis patients in the initial (MAT negative) or convalescent (MAT positive) phases of the disease. The evolution of TF quantification at the initial and convalescent phases of the disease on individual patients is represented at the panel B. Sera from healthy donors were used as control. The bars represent the means of individual samples ± standard deviation.

Next, we wished to analyze the coagulative state of plasma samples from *Leptospira* patients. We measured TF concentrations in samples from initial phase (MAT negative) and convalescent phase (MAT positive) from the same patients. We found the highest medium TF concentrations in MAT negative (134.79±54.98 pg/mL) and MAT positive (97.33±30.66 pg/mL) patients compared to the levels in sera from or healthy individuals (below detectable values) (Fig 6A). Although there is no significant statistical difference between the two groups, we also observed that there is a general trend showing that TF levels tend to decline during disease progression (Fig 6B). As TF activity is regulated by tissue factor pathway inhibitor (TFPI), we determined the concentration of TFPI in these samples. There were no significant differences of TFPI levels in the two patients groups (MAT negative 31.78±5.48 ng/mL; MAT positive 29.66±7.84 ng/mL) compared to control (28.34±1.97 ng/mL) (Fig 6C). These results further indicate the pro-coagulant state during leptospirosis.

### Pro-coagulant MVs are shed upon PBMCs stimulation with *Leptospira* and are increased in human leptospirosis serum samples

**Fig 7:**
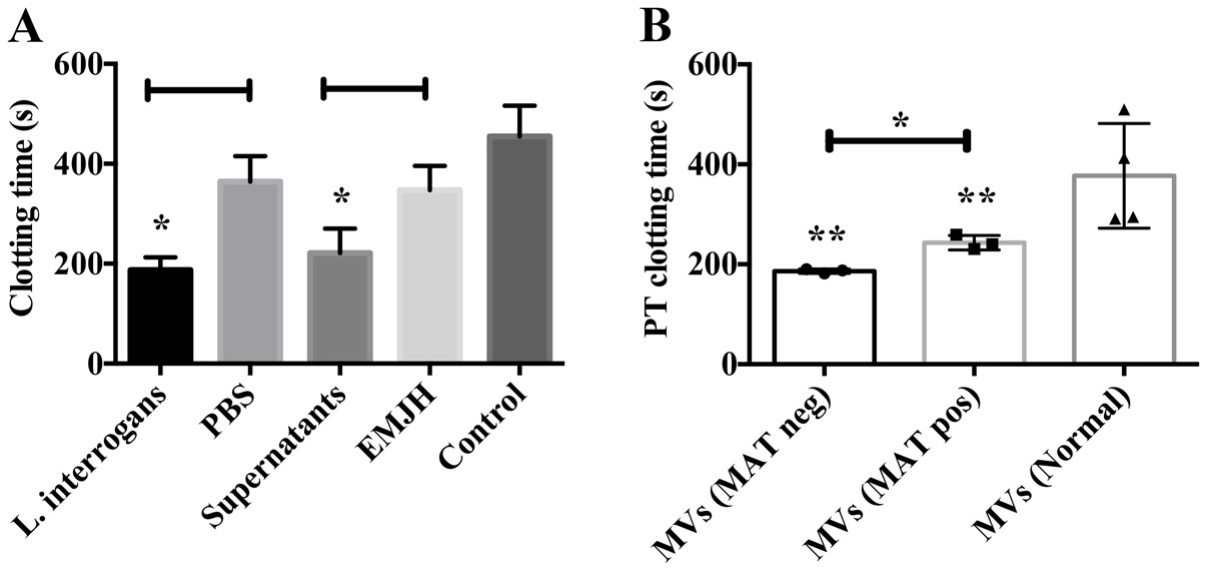
Analysis of the pro-coagulant activity of MVs derived from monocytes or leptospirosis patients’ sera. (A) PBMCs were purified from human blood, and stimulated with live leptospires or its culture supernatants for 16 h at 37°C. Cells stimulated with PBS or EMJH culture medium were used as controls. MVs shed to the supernatants were purified, 5 μL were added to human plasma, and the recalcification times were determined in a coagulometer. Control: recalcification time of plasma with no additions. (B) MVs isolated from pooled sera (5 μL) were added to human plasma, recalcified in the presence of the PT reagent and clotting times were determined. Pooled sera from healthy donors were used as control. The bars represent the means ± standard deviation of three replicates and are representative of two independent experiments. *P > 0.005 and **P > 0.05.

MVs are membrane vesicles that are released by many cell types and have been recently implicated in a number of biological processes, such as hemostasis, inflammation and host defense, contributing to the pathogenesis of many diseases [28, 29]. We therefore decided to determine the coagulative state of MVs released from purified human PBMCs stimulated with leptospires or culture supernatants. The PBMCs-derived MVs were added to human plasma, and the recalcification clotting times were determined. Both bacterial cells and secreted products were able to stimulate the release of pro-coagulant MVs by monocytes, as observed by the decreased recalcification clotting times when compared to the controls (PBMCs stimulated with buffer or EMJH alone) (Fig 7A).

We also analyzed the coagulative state from MVs purified from leptospirosis patients’ sera. MVs were added to plasma, and the recalcification clotting times with or without PT reagent were determined. Although no significant changes were observed for the recalcification time (S2 Fig), a decrease in the clotting times was observed for both MAT negative and MAT positive samples in the presence of PT reagent (Fig 7B).

**Fig 8:**
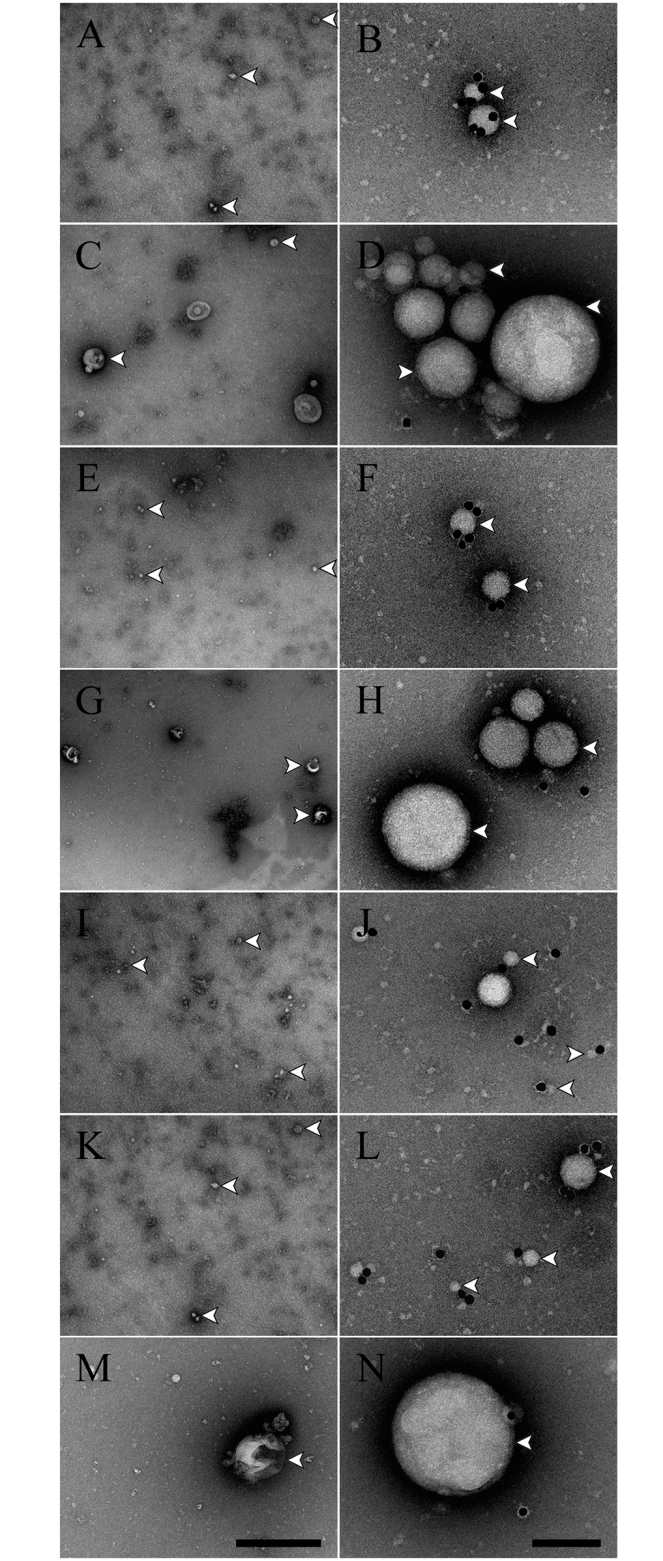
Characterization of the MVs derived from monocytes or leptospirosis patients’ sera. MVs derived from stimulated PBMCs (*panels A-H*) or purified from human sera (*panels I-N*) were incubated with a gold-labelled antibody against TF, processed by negative staining and analysed in a transmission electron microscope at two different magnifications. PBMCs-derived MVs were purified from cells stimulated with *L*. *interrogans* (*panels A and B*), PBS (*panels C and D*), leptospires culture supernatants (*panels E and F*) or EMJH fresh culture medium (*panels G and H*). MVs were purified from human leptospirosis patients sera at the early phase (*panels I and J*) or convalescent phase (*panels K and L*) of the disease. MVs purified from nomal human sera were used as controls (*panels M and N*). Scale bars represent 500 nm (left) or 100 nm (right). White arrowheads indicate some of the MVs present on each image.

By immunoelectron microscopy, we observed that MVs derived from PBMCs stimulated with leptospires or its culture supernatants are smaller in size and present higher exposure of TF than the controls stimulated with PBS or EMJH (Fig 8A–8H). A similar pattern was seen for patients-derived MVs, where a higher number of small nanovesicles harboring more TF than normal control were found for both early and convalescent-phases of the disease (Fig 8I–8N). Together, our results indicate higher activity of the extrinsic pathway of coagulation, suggesting that during leptospirosis, pro-coagulant MVs harboring TF are shed from activated monocytes.

## Discussion

As soon as leptospires enter the host, mainly through skin or mucosa, the pathogen starts to disseminate throughout the body. Given the extremely high number of bacteria in the circulation [30], the host immune system can ultimately trigger exaggerated systemic responses leading to severe complications. Such complications can be caused by a massive release of cytokines or dysfunction of the coagulation system [31]. In cases of excessive inflammation, leptospirosis patients may then suffer from high fever and tubulo-interstitial nephritis with interstitial edema and containing massive immune cells infiltrates [32, 33], whereas an impairment of the coagulation system is often associated with severe bleeding complications.

Coagulation is a tightly controlled chain of events that can be initiated via an extrinsic or intrinsic pathway. While the extrinsic pathway is essential for normal clot formation, the intrinsic, also known as the contact system, plays a secondary role in hemostasis. However, the latter pathway is involved in triggering inflammatory reactions through the formation of the pro-inflammatory peptide BK [14, 34–36]. Miragliotta and colleagues [37] have previously shown that monocytes incubated with *Leptospira* were able to shorten the recalcification time of normal plasma, but were ineffective in factor VII deficient plasma, suggesting the generation of TF-like activity. Accordingly, our results show an induction of TF-mediated pro-coagulant activity by *Leptospira* and their secreted products under *in vitro* conditions and increased TF levels in the sera of patients suffering from leptospirosis. These findings point to an important role of TF in the induction of the pro-coagulant state during leptospirosis. Moreover, our data further demonstrate that MVs isolated from patients samples have pro-coagulant properties, exhibiting different size characteristics and harboring more TF than healthy controls. Both results support the notion that systemic activation of coagulation can take place during leptospirosis and may eventually lead to severe coagulation disturbaces including DIC, though DIC occurence is controversial in leptospirosis [23, 24, 38].

Within the last two decades there are many studies reporting an involvement of the contact system in the pathophysiology of sepsis [39]. It has been shown that the assembly and activation of the contact system at the surface of human pathogens, such as *Streptococcus pyogenes*, *Escherichia coli*, and *Salmonella* spp., can induce a massive release of BK, that in turn may lead to increased vascular permeability and plasma leakage and is followed by bacterial dissemination and massive inflammatory reactions [39–41]. These findings are in line with our results showing that leptospires provide a surface that allows the recruitment and activation of the contact system. The release of BK during leptospirosis may therefore constitute an important mechanism that considerably contributes to inflammatory reactions during the disease. Under systemic conditions this may contribute to some of the leptospirosis symptoms like fever, pain and hypotension. In addition, inflammation of blood vessels and vasculitis are conditions often associated to leptospirosis [42], and thus our results provide a plausible explanation for the molecular mechanisms behind these complications.

Apart from triggering inflammatory reactions, kinins can induce the release of tissue-type plasminogen activator (tPA) from endothelial cells [43, 44]. Our previous studies have shown that elevated tPA levels can be measured in human leptospirosis, especially in the early phase of the disease [17]. As BK is an early inflammatory mediator, these findings support the idea that BK release could be involved in the mobilization of tPA during the hematogenous leptospirosis phase. Thus our results can provide an explanation for the hemostatic imbalance of leptospirosis by fibrinolytic activation. Bleeding disorders are common complications in severe leptospirosis and there are many evidences emphasizing that activation of coagulation, impaired anticoagulation, and activation of fibrinolysis play a significant role in these processes [17, 20–24].

Although incipient, studies revealing mechanisms by which leptospires interfere with host immune responses and hemostasis have enlightened our understanding of *Leptospira* host-pathogen interactions in the last few years [19, 45]. Our results further contribute to an increased knowledge of the pathophysiological mechanisms in leptospirosis and uncover new mechanisms by which this pathogen interferes with the host response and gives rise to pathological signs. Here we show that both pathways of the coagulation system are modulated by leptospires and this may open new directions for diagnosis and treatment in the severe manifestations of leptospirosis.

## Supporting Information

S1 Fig The influence of *Leptospira* culture supernatants in human blood and plasma coagulative state. Culture supernatants of virulent *L*. *interrogans* serovar Copenhageni L1-130, culture-attenuated *L*. *interrogans* serovar Copenhageni and saprophytic *L*. *biflexa* were incubated with citrated human blood at increasing incubation intervals (0.5, 1, 2 or 4 h) (A) or with human plasma for 30 min (B) and the recalcification clotting times were determined in a coagulometer. Fresh culture medium was used as control. The bars represent the means ± standard deviation of four replicates and are representative of three independent experiments.
(DOCX)

S2 Fig Effects of sera-derived microvesicles in human plasma coagulative state. MVs isolated from pooled leptospirosis patients’ sera were added to human plasma and the recalcification clotting times were determined. Pooled sera from healthy donors were used as control. The bars represent the means ± standard deviation of individual measures and are representative of two independent experiments.
(DOCX)

## References

[1] Bharti AR, Nally JE, Ricaldi JN, Matthias MA, Diaz MM, Lovett MA, et al Leptospirosis: a zoonotic disease of global importance. The Lancet Infectious diseases. 2003;3(12):757–71. . 14652202 10.1016/s1473-3099(03)00830-2

[2] Levett PN. Leptospirosis. Clin Microbiol Rev. 2001;14(2):296–326. 10.1128/CMR.14.2.296-326.2001 11292640 PMC88975

[3] Faine S, Adler B, Bolin C, Perolat P. *Leptospira* and Leptospirosis. Second ed Melbourne, Australia: MediSci; 1999 p. 259.

[4] Plank R, Dean D. Overview of the epidemiology, microbiology, and pathogenesis of *Leptospira* spp. in humans. Microbes and infection / Institut Pasteur. 2000;2(10):1265–76. . 11008116 10.1016/s1286-4579(00)01280-6

[5] Ko AI, Goarant C, Picardeau M. *Leptospira*: the dawn of the molecular genetics era for an emerging zoonotic pathogen. Nature reviews Microbiology. 2009;7(10):736–47. Epub 2009/09/17. 10.1038/nrmicro2208 . 19756012 PMC3384523

[6] Vinetz JM. Leptospirosis. Curr Opin Infect Dis. 2001;14(5):527–38. . 11964872 10.1097/00001432-200110000-00005

[7] Adler B, Lo M, Seemann T, Murray GL. Pathogenesis of leptospirosis: the influence of genomics. Veterinary microbiology. 2011;153(1–2):73–81. Epub 2011/03/29. 10.1016/j.vetmic.2011.02.055 . 21440384

[8] Cinco M. New insights into the pathogenicity of leptospires: evasion of host defences. The new microbiologica. 2010;33(4):283–92. Epub 2011/01/11. . 21213586

[9] Evangelista KV, Coburn J. *Leptospira* as an emerging pathogen: a review of its biology, pathogenesis and host immune responses. Future microbiology. 2010;5(9):1413–25. 10.2217/fmb.10.102 20860485 PMC3037011

[10] Cicala C, Cirino G. Linkage between inflammation and coagulation: an update on the molecular basis of the crosstalk. Life Sci. 1998;62(20):1817–24. 9600323 10.1016/s0024-3205(97)01167-3

[11] Mackman N. Role of tissue factor in hemostasis and thrombosis. Blood cells, molecules & diseases. 2006;36(2):104–7. 10.1016/j.bcmd.2005.12.008 . 16466951

[12] Colman RW. Biologic activities of the contact factors *in vivo*—potentiation of hypotension, inflammation, and fibrinolysis, and inhibition of cell adhesion, angiogenesis and thrombosis. Thromb Haemost. 1999;82(6):1568–77. 10613636

[13] Kitchens CS. The contact system. Arch Pathol Lab Med. 2002;126(11):1382–6. . 12421145 10.5858/2002-126-1382-TCS

[14] Frick IM, Bjorck L, Herwald H. The dual role of the contact system in bacterial infectious disease. Thrombosis and haemostasis. 2007;98(3):497–502. . 17849037

[15] Tapper H, Herwald H. Modulation of hemostatic mechanisms in bacterial infectious diseases. Blood. 2000;96(7):2329–37. . 11001879

[16] Vieira ML, Vasconcellos SA, Goncales AP, de Morais ZM, Nascimento AL. Plasminogen acquisition and activation at the surface of *Leptospira* species lead to fibronectin degradation. Infect Immun. 2009;77(9):4092–101. 10.1128/IAI.00353-09 19581392 PMC2738053

[17] Vieira ML, Alvarez-Flores MP, Kirchgatter K, Romero EC, Alves IJ, de Morais ZM, et al Interaction of *Leptospira interrogans* with human proteolytic systems enhances dissemination through endothelial cells and protease levels. Infect Immun. 2013;81(5):1764–74. 10.1128/IAI.00020-13 23478319 PMC3648023

[18] Vieira ML, de Morais ZM, Vasconcellos SA, Romero EC, Nascimento AL. In vitro evidence for immune evasion activity by human plasmin associated to pathogenic *Leptospira interrogans*. Microb Pathog. 2011;51(5):360–5. 10.1016/j.micpath.2011.06.008 . 21802507

[19] Vieira ML, Nascimento AL. Interaction of spirochetes with the host fibrinolytic system and potential roles in pathogenesis. Critical reviews in microbiology. 2015:1–15. 10.3109/1040841X.2014.972336 . 25914944

[20] Sitprija V, Pipatanagul V, Mertowidjojo K, Boonpucknavig V, Boonpucknavig S. Pathogenesis of renal disease in leptospirosis: Clinical and experimental studies. Kidney international. 1980;17(6):827–36. Epub 1980/06/01. . 7412114 10.1038/ki.1980.95

[21] Edwards CN, Nicholson GD, Hassell TA, Everard CO, Callender J. Thrombocytopenia in leptospirosis: the absence of evidence for disseminated intravascular coagulation. The American journal of tropical medicine and hygiene. 1986;35(2):352–4. Epub 1986/03/01. . 3953949 10.4269/ajtmh.1986.35.352

[22] De Francesco Daher E, Oliveira Neto FH, Ramirez SM. Evaluation of hemostasis disorders and anticardiolipin antibody in patients with severe leptospirosis. Revista do Instituto de Medicina Tropical de Sao Paulo. 2002;44(2):85–90. Epub 2002/06/06. . 12048545 10.1590/s0036-46652002000200006

[23] Chierakul W, Tientadakul P, Suputtamongkol Y, Wuthiekanun V, Phimda K, Limpaiboon R, et al Activation of the coagulation cascade in patients with leptospirosis. Clinical infectious diseases: an official publication of the Infectious Diseases Society of America. 2008;46(2):254–60. Epub 2008/01/04. 10.1086/524664 . 18171258

[24] Wagenaar JF, Goris MG, Partiningrum DL, Isbandrio B, Hartskeerl RA, Brandjes DP, et al Coagulation disorders in patients with severe leptospirosis are associated with severe bleeding and mortality. Trop Med Int Health. 2010;15(2):152–9. 10.1111/j.1365-3156.2009.02434.x . 20002620

[25] Persson K, Morgelin M, Lindbom L, Alm P, Bjorck L, Herwald H. Severe lung lesions caused by *Salmonella* are prevented by inhibition of the contact system. J Exp Med. 2000;192(10):1415–24. 11085744 10.1084/jem.192.10.1415 PMC2193180

[26] Bengtson SH, Phagoo SB, Norrby-Teglund A, Pahlman L, Morgelin M, Zuraw BL, et al Kinin receptor expression during *Staphylococcus aureus* infection. Blood. 2006;108(6):2055–63. 10.1182/blood-2006-04-016444 16735595 PMC1895540

[27] Bober M, Enochsson C, Collin M, Morgelin M. Collagen VI is a subepithelial adhesive target for human respiratory tract pathogens. Journal of innate immunity. 2010;2(2):160–6. 10.1159/000232587 . 20375633

[28] Anderson HC, Mulhall D, Garimella R. Role of extracellular membrane vesicles in the pathogenesis of various diseases, including cancer, renal diseases, atherosclerosis, and arthritis. Laboratory investigation; a journal of technical methods and pathology. 2010;90(11):1549–57. 10.1038/labinvest.2010.152 . 20805791

[29] Yuana Y, Sturk A, Nieuwland R. Extracellular vesicles in physiological and pathological conditions. Blood reviews. 2013;27(1):31–9. 10.1016/j.blre.2012.12.002 . 23261067

[30] Agampodi SB, Matthias MA, Moreno AC, Vinetz JM. Utility of quantitative polymerase chain reaction in leptospirosis diagnosis: association of level of leptospiremia and clinical manifestations in Sri Lanka. Clinical infectious diseases: an official publication of the Infectious Diseases Society of America. 2012;54(9):1249–55. 10.1093/cid/cis035 22354922 PMC3404689

[31] Reis EA, Hagan JE, Ribeiro GS, Teixeira-Carvalho A, Martins-Filho OA, Montgomery RR, et al Cytokine response signatures in disease progression and development of severe clinical outcomes for leptospirosis. PLoS neglected tropical diseases. 2013;7(9):e2457 10.1371/journal.pntd.0002457 24069500 PMC3777885

[32] Yang CW. Leptospirosis renal disease: understanding the initiation by Toll-like receptors. Kidney international. 2007;72(8):918–25. 10.1038/sj.ki.5002393 . 17687261

[33] Yang CW, Wu MS, Pan MJ. Leptospirosis renal disease. Nephrology, dialysis, transplantation: official publication of the European Dialysis and Transplant Association—European Renal Association. 2001;16 Suppl 5:73–7. . 11509689 10.1093/ndt/16.suppl_5.73

[34] Nickel KF, Renne T. Crosstalk of the plasma contact system with bacteria. Thrombosis research. 2012;130 Suppl 1:S78–83. 10.1016/j.thromres.2012.08.284 . 23026673

[35] Renne T. The procoagulant and proinflammatory plasma contact system. Seminars in immunopathology. 2012;34(1):31–41. 10.1007/s00281-011-0288-2 . 21858560

[36] Opal SM. Interactions between coagulation and inflammation. Scandinavian journal of infectious diseases. 2003;35(9):545–54. 10.1080/00365540310015638 . 14620133

[37] Miragliotta G, Barone G, Ricci G, Dimonte D, Marcuccio C, Fumarola D. In vitro effect of *Leptospira icterohaemorrhagiae* on human mononuclear leukocytes: comparison of virulent with non virulent strain. G Batteriol Virol Immunol. 1982;75(1–6):3–8. . 7187349

[38] Yang HL, Jiang XC, Zhang XY, Li WJ, Hu BY, Zhao GP, et al Thrombocytopenia in the experimental leptospirosis of guinea pig is not related to disseminated intravascular coagulation. BMC infectious diseases. 2006;6:19 10.1186/1471-2334-6-19 16451735 PMC1434752

[39] Herwald H, Morgelin M, Bjorck L. Contact activation by pathogenic bacteria: a virulence mechanism contributing to the pathophysiology of sepsis. Scandinavian journal of infectious diseases. 2003;35(9):604–7. . 14620142 10.1080/00365540310016268

[40] Ben Nasr A, Herwald H, Sjobring U, Renne T, Muller-Esterl W, Bjorck L. Absorption of kininogen from human plasma by *Streptococcus pyogenes* is followed by the release of bradykinin. The Biochemical journal. 1997;326 (Pt 3):657–60. 9307013 PMC1218718

[41] Herwald H, Morgelin M, Olsen A, Rhen M, Dahlback B, Muller-Esterl W, et al Activation of the contact-phase system on bacterial surfaces—a clue to serious complications in infectious diseases. Nature medicine. 1998;4(3):298–302. . 9500602 10.1038/nm0398-298

[42] Medeiros Fda R, Spichler A, Athanazio DA. Leptospirosis-associated disturbances of blood vessels, lungs and hemostasis. Acta tropica. 2010;115(1–2):155–62. 10.1016/j.actatropica.2010.02.016 . 20206112

[43] Brown NJ, Nadeau JH, Vaughan DE. Selective stimulation of tissue-type plasminogen activator (t-PA) in vivo by infusion of bradykinin. Thrombosis and haemostasis. 1997;77(3):522–5. . 9066005

[44] van den Eijnden-Schrauwen Y, Kooistra T, de Vries RE, Emeis JJ. Studies on the acute release of tissue-type plasminogen activator from human endothelial cells in vitro and in rats in vivo: evidence for a dynamic storage pool. Blood. 1995;85(12):3510–7. . 7780137

[45] Chirathaworn C, Kongpan S. Immune responses to *Leptospira* infection: roles as biomarkers for disease severity. The Brazilian journal of infectious diseases: an official publication of the Brazilian Society of Infectious Diseases. 2014;18(1):77–81. 10.1016/j.bjid.2013.08.002 . 24275371 PMC9425245

