# Supplementary material for: Modulation of Hemostatic and Inflammatory Responses by Leptospira Spp
Source: PLoS Negl Trop Dis. 2016 May 11;10(5):e0004713. doi: 10.1371/journal.pntd.0004713 (PMC4864083; doi:10.1371/journal.pntd.0004713)
Supplement: S1 Fig — Culture supernatants of virulent L. interrogans serovar Copenhageni L1-130, culture-attenuated L. interrogans serovar Copenhageni and saprophytic L. biflexa were incubated with citrated human blood at increasing incubation intervals (0.5, 1, 2 or 4 h) (A) or with human plasma for 30 min (B) and the recalcification clotting times were determined in a coagulometer. Fresh culture medium was used as control. The bars represent the means ± standard deviation of four replicates and are representative of three independent experiments. (DOCX) [file pntd.0004713.s001.docx]

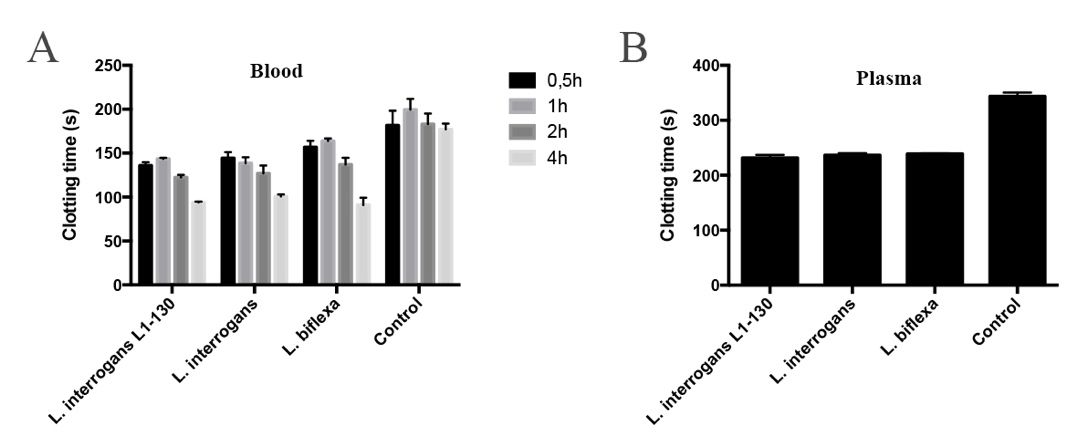


**S1 Fig.** **The influence of *Leptospira* culture supernatants in human blood and plasma coagulative state.** Culture supernatants of virulent *L. interrogans* serovar Copenhageni L1-130, culture-attenuated *L. interrogans* serovar Copenhageni and saprophytic *L. biflexa* were incubated with citrated human blood at increasing incubation intervals (0.5, 1, 2 or 4 h) (A) or with human plasma for 30 min (B) and the recalcification clotting times were determined in a coagulometer. Fresh culture medium was used as control. The bars represent the means ± standard deviation of four replicates and are representative of three independent experiments.
