# Supplementary material for: Modulation of Hemostatic and Inflammatory Responses by Leptospira Spp
Source: PLoS Negl Trop Dis. 2016 May 11;10(5):e0004713. doi: 10.1371/journal.pntd.0004713 (PMC4864083; doi:10.1371/journal.pntd.0004713)
Supplement: S2 Fig — MVs isolated from pooled leptospirosis patients’ sera were added to human plasma and the recalcification clotting times were determined. Pooled sera from healthy donors were used as control. The bars represent the means ± standard deviation of individual measures and are representative of two independent experiments. (DOCX) [file pntd.0004713.s002.docx]

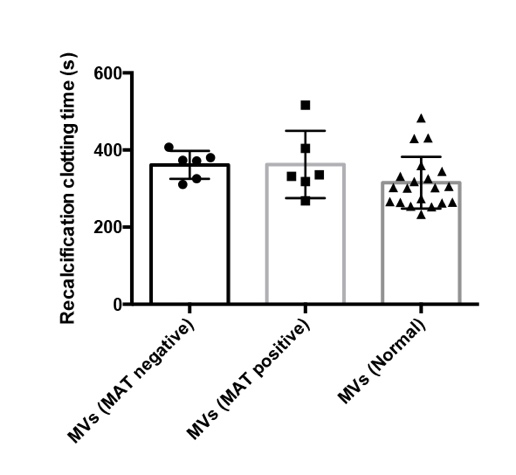


**S2 Fig. Effects of sera-derived microvesicles in human plasma coagulative state.** MVs isolated from pooled leptospirosis patients’ sera were added to human plasma and the recalcification clotting times were determined. Pooled sera from healthy donors were used as control. The bars represent the means ± standard deviation of individual measures and are representative of two independent experiments.
